# Mortality drives production dynamics of Atlantic cod through 1100 years of commercial fishing

**DOI:** 10.1126/sciadv.adt4782

**Published:** 2025-02-05

**Authors:** Steven E. Campana, George Hambrecht, Nicole Misarti, Habiba Moshfeka, Mary Efird, Sara M. Schaal, Guðbjörg Ásta Ólafsdóttir, Ragnar Edvardsson, Árni Daníel Júlíusson, Einar Hjörleifsson, Frank J. Feeley, Grace Cesario, Lilja Björk Palsdóttir

**Affiliations:** ^1^ Life and Environmental Sciences, University of Iceland, Reykjavik, Iceland.; ^2^ Department of Anthropology, University of Maryland, College Park, MD 20742, USA.; ^3^ Water and Environmental Research Center, University of Alaska Fairbanks, Fairbanks, AK 99775, USA.; ^4^ Department of Fisheries, University of Alaska Fairbanks, Fairbanks, AK 99775, USA.; ^5^ Department of Marine and Environmental Sciences, Northeastern University, Nahant, MA 01908, USA.; ^6^ Research Centre of the Westfjords, University of Iceland, Bolungarvik, Iceland.; ^7^ Stefansson Arctic Institute, Akureyri, Iceland.; ^8^ Marine and Freshwater Research Institute, Hafnarfjörður, Iceland.; ^9^ Queens College, City University of New York, Flushing, NY 11367, USA.; ^10^ Department of Archaeology, University of Iceland, Reykjavik, Iceland.; ^11^ Háskólinn á Hólum, Hólar University, Hólar, Iceland.

## Abstract

Most edible fish species have been fished for centuries or millennia, leaving little record or understanding of their population responses prior to human impact and thus no baseline for population conservation. Here, we reconstruct the population dynamics of Atlantic cod, one of the world’s most harvested fish species, from the pristine state during the Viking era through more than 1100 years of fishing. Analysis of cod otoliths recovered during archaeological excavations of Icelandic fish processing sites revealed that cod in the 10th to 12th centuries were 25% larger and up to 300% older than modern, despite slower density-dependent growth rates attributed to the sixfold increase in abundance. Fishing mortality came to dominate a time-invariant natural mortality rate and other population characteristics after the 14th century, with minimal evidence of environmental effects at the century scale. Despite the absence of catch records and surveys, biological reference points based on pristine fish populations are now possible where otolith collections are available.

## Teaser

Fishing in the Viking era did not affect the Icelandic cod population, but foreign markets in the 14th century changed everything.

## INTRODUCTION

Birth, growth, and mortality are the key demographic rates that govern the abundance and biomass of all living organisms. In the absence of a precise balance between mortality and birth (or replacement) rates, density dependence constrains the rate of these processes so as to avoid unchecked exponential increases or decreases in population abundance. Malthus referred to the “positive checks” of famine and disease in his human-centric view of population regulation (*1*), but modern views of density dependence in vertebrate species typically manifest as constraints on reproductive output and mortality, which, in turn, come about through competition for food and habitat, increased predation, reduced fecundity, and delayed age at maturity (*2*). Together, estimates of demographic rates and their density dependence form the basis for most vertebrate population dynamics models and underlie the biological reference points that govern fisheries management and wildlife conservation efforts around the world (*3*).

The abundance and population dynamics of most mammals can be directly monitored, often through observation at the individual level (*4*). In contrast, assessment of marine fish populations is seldom possible at the individual level and is instead derived from modeling of extensive but indirect fishery measures such as catch, catch rate, and age composition collected during harvesting or management. Using fisheries data collected over the past century, the population rates and density-dependent responses of overharvested, low-abundance fish populations have been reasonably well documented (*5*, *6*). Ironically, the absence of fisheries data has left the population dynamics and characteristics of unfished populations virtually unknown; most edible fish species have been fished for centuries or millennia, leaving little record or understanding of their population responses during their pristine phase. In the rare examples that have been documented, lightly exploited fish populations tend to be dominated by abundant large and old animals, low total mortality rates, and sustainable recruitment levels (*7*–*9*), all of which reduce fluctuations in abundance, increase population resilience, and promote ecosystem stability (*10*, *11*). However, in a classic example of “the shifting baseline principle” (*12*), many population assessments have increasingly abandoned any attempt to refer to the original abundance, size, and age structures of fish populations for setting more accurate biological reference points, in large part because the original parameters can no longer be estimated (*13*).

Atlantic cod (*Gadus morhua*) is an apex predator and keystone species of the North Atlantic Ocean and has been the target of concerted fishing effort for more than 1100 years (*14*). None of the existing 22 cod stocks have catch or abundance records dating to the onset of fishing, and all intermix with adjacent populations, thus complicating even modern fisheries assessments. An exception is the large Icelandic cod population, which is relatively isolated geographically and genetically from both European and North American populations (*15*), with a known onset of fishing activity associated with first settlement by Norse settlers and Vikings around the year 874 (*16*).

Atlantic cod fishing by Icelandic Norse communities in the Viking age was targeted at migratory spring spawners, conducted from small open boats equipped with oars and sails. The catch was flat dried and traded among coastal and inland settlements, a practice common to early Norse settlements in other countries (*17*–*19*). By the medieval period, Iceland had entered into trade with Europe, with stockfish (a highly standardized form of round-dried cod) emerging as a major export to feed Europe’s burgeoning urban populations (*14*, *20*). Stockfish demands were initially met by traditional fishing and dried fish production in Iceland and regulated by the Danish crown. In the 14th century, technological innovations introduced by English fishermen led to intensified exploitation of Iceland’s cod fishery (*21*, *22*). The large, decked fishing vessels used by the English and other foreign fishermen could hold more fish and exploit deeper waters than the open sailed rowboats used by local Icelandic fishermen *(**22*). In the 15th to 16th centuries, European nations such as the English and Dutch increasingly bypassed the Danish trade restrictions of the North Atlantic and sent their own fishing fleets to the Icelandic fishing grounds (*23*–*27*). Icelandic fishermen did not adopt decked ships until the mid-19th century and engine-powered boats and trawlers in the early 20th century, when catches more than tripled (*28*).

Archaeological excavations of middens associated with discarded household and community wastes have been used to reconstruct many aspects of Icelandic history, but some of the most promising have come from large-scale fishing sites (*29*, *30*). The relationship between dried fish production and consumption sites is defined quite clearly in the zooarchaeological record of Iceland, especially in the case of cod. The coastal production site assemblages are most often dominated by the cranial elements of the fish, whereas consumption sites are dominated by the vertebral elements (*17*, *31*). This reflects the fact that dried cod products were headless, and the processing discarded the cranial elements at the production sites, whereas consumer sites are dominated by the vertebrae that were left in the dried product. Otoliths, the calcified pair of acellular and metabolically inert earstones found in all fishes, are a cranial element and are thus found at dried fish production sites. Although the annular growth bands found in the otolith provide the basis for modern growth, age composition, and mortality rate estimates in most global fish population assessments (*32*), otoliths recovered from archaeological sites in Iceland and elsewhere have typically only been used to estimate mean growth rate or determine the season of capture (*33*, *34*). Yet, with minimal assumptions and given a pristine population, catch, relative abundance, and the complicated balance between natural and fishing mortality can all be estimated with otoliths without the use of fishing records or surveys. In a manner similar to paleoclimate reconstructions from the calcium carbonate matrix of corals, the stable isotope composition of the calcium carbonate matrix of otoliths can be used to put an environmental context around the period of otolith accretion (*35*). As temperature and food are well-known modifiers of fish growth and demography, the analysis of stable oxygen isotopes in otolith growth bands can be used to control for concurrent changes in water temperature. Interpretation of the carbon isotopic signature is less straightforward in otoliths than in muscle tissue (*36*) but can still be used to identify major shifts in metabolic rate and the food web, which could influence growth.

Here, we develop a protocol for reconstructing virtually the entire suite of key population dynamics parameters for a fish population using only otolith-derived information. We then apply this approach to collections of archaeologically excavated and dated (hereafter referred to as “historical”) otoliths from Iceland to provide a unique time series of the population dynamics of one of the world’s most harvested fish species, from the initial Norse and Viking colonization through more than 1100 years of increasingly intensive fishing. Our objective was to determine how and when the cod population responded to the fishing pressure and the role of the environment in the population’s historical ecology and demography.

## RESULTS

### Archaeological context

**Fig. 1.**
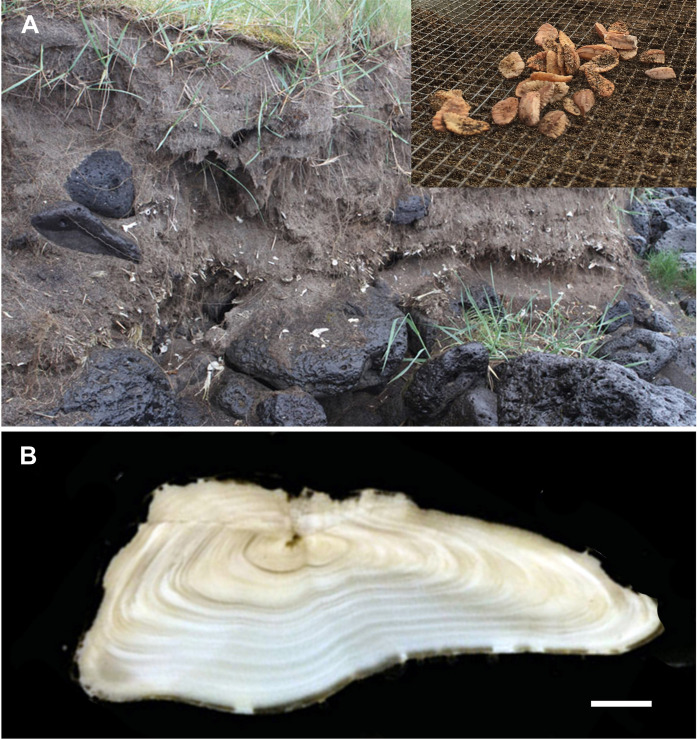
Otolith sampling and quality. (**A**) Sampling site in Gufuskálar showing fish bones and otoliths in situ. Image credit: F.J.F., City University of New York. Insert shows 15th century cod otoliths from the Breiðavik site. Image credit: G.A.O., University of Iceland. (**B**) Annual growth bands visible in a transverse section of an 11-year-old cod otolith from the 15th century. Only those sections where the diameter was intact and annual growth increments were visible were used for fish length and age estimation. Scale bar, 1 mm. Image credit: S.E.C., University of Iceland.

The 12 archaeological sites used in this study dated from the earliest settlement of Iceland in the second half of the 9th century through to the 19th century (fig. S1 and table S1). All sites represented fish drying (production) locations, intended for either domestic consumption or export, because all were dominated by cranial elements (such as otoliths) rather than vertebral elements (Fig. 1A). The sites included coastal farms directly involved in fishing and fish drying, small dedicated coastal fishing stations manned by locals, and larger fishing stations used as production sites for export. All fishing is believed to have been done with hook and line, and there is no evidence of size-selective discarding or catch restrictions until at least the 19th century.

A total of 651 cod sagittal otoliths were examined (Table 1), of which 629 were suitable for either length estimation (*N* = 589), age determination (*N* = 605), or isotopic assay (*N* = 335). Most of the otoliths were associated with the 15th century (31%), but 18% derived from the 10th to 14th centuries, and 51% from the 16th century and later. Otolith quality was more strongly associated with site rather than age, with some of the oldest otoliths so well preserved as to be mistaken for modern (Fig. 1B).

**Table 1.** Sampling site information.

Site	Site code	Region	Centuries	*N* lengths	*N* ages	*N* isotopes
Akurvík	AKU	NW	12 to 13	44	48	48
Breiðavík	BRV	W	10, 14 to 19	356	362	127
Gjögur	GJ	NW	15	3	3	3
Grænagerði	GNR	NW	11	14	14	13
Gufuskálar	GFS	W	15	53	54	54
Kotið	KOT	NW	10	6	6	6
Kollsvík	KOV	W	18 to 19	48	48	0
Næfurstaðir	NFS	NW	10	8	12	11
Strákey	STE	NW	16	12	12	11
Útskálar	UTS	W	19	5	6	6
Vatnskot	VTN	NW	10 to 11	40	40	38

### Length composition of historical and modern cod

**Fig. 2.**
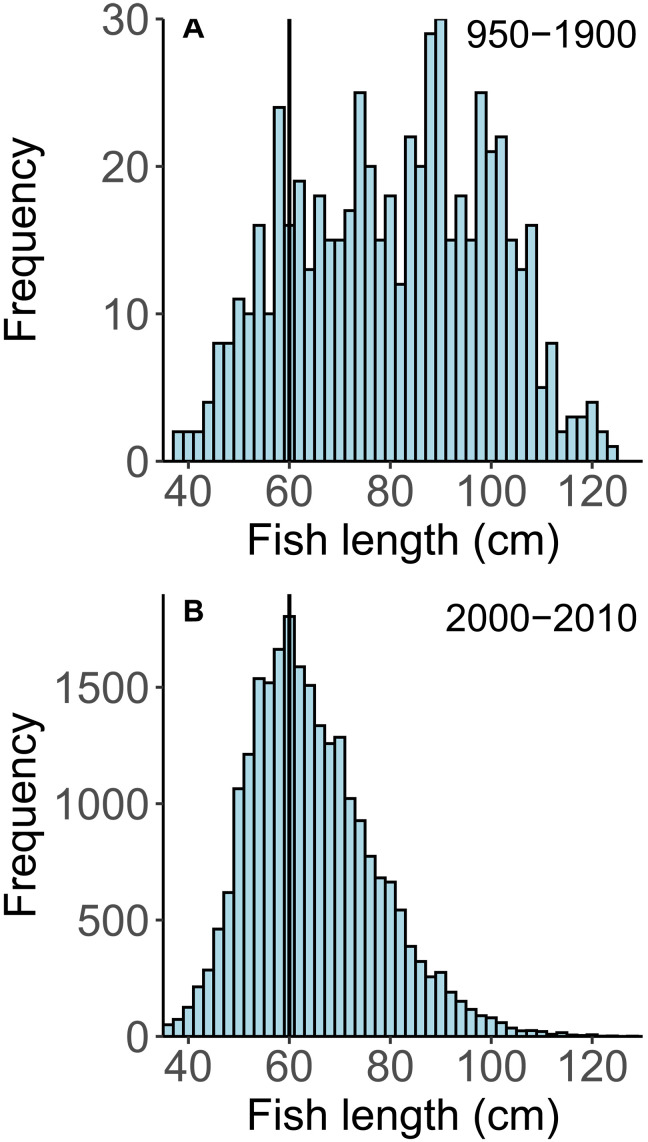
Length composition of archaeological and modern cod. The solid vertical line indicates the modal length of modern cod (= 60 cm) in the sampling region. (**A**) Cod lengths estimated from the size of otoliths recovered from the historical sample sites. (**B**) Cod lengths recorded in the 2000 to 2010 commercial hook and line fishery in the study area.

Annual otolith growth in cod is tightly coupled to growth in fish length, allowing the length of cod to be accurately estimated based only on otolith dimensions (fig. S2; *P* < 0.001, *R*^2^ = 0.95; *n* = 114). Reconstructed lengths of historical cod ranged from 38 to 124 cm with a mean length of 81.1 cm (*n* = 581) (Fig. 2A). In contrast, cod lengths from the modern (years 2000 to 2010) hook and line fishery in the study area were 20% smaller (mean of 65.1 cm; range of 32 to 160 cm; *n* = 24,324) (Fig. 2B). The minimum size of 32 cm observed in the modern hook and line fishery was very similar to the 38 cm minimum estimated for the historical cod, suggesting that there was no appreciable bias against smaller otoliths in the archaeological excavations. Nevertheless, 79% of the historical cod exceeded the mean length of the modern cod caught with hooks, and 86% exceeded that caught in the trawl survey, which more closely represents the actual size composition of the population (fig. S3). Given that modern hook and line fishing gear is designed to capture the largest fish available and given the less-developed nature of the early fishing gear, the difference between historical and modern size composition is probably even more skewed than is shown.

Modern cod from the western region (W) tended to be 10 cm larger than those from the northwestern region (NW) (63.1 cm versus 53.3 cm, respectively). A similar regional size differential existed in the historical otoliths, where W otoliths averaged 83.2 cm and NW otoliths averaged 73.3 cm. For this reason, region was included as a factor in all analyses involving length, growth, or isotopic composition.

**Fig. 3.**
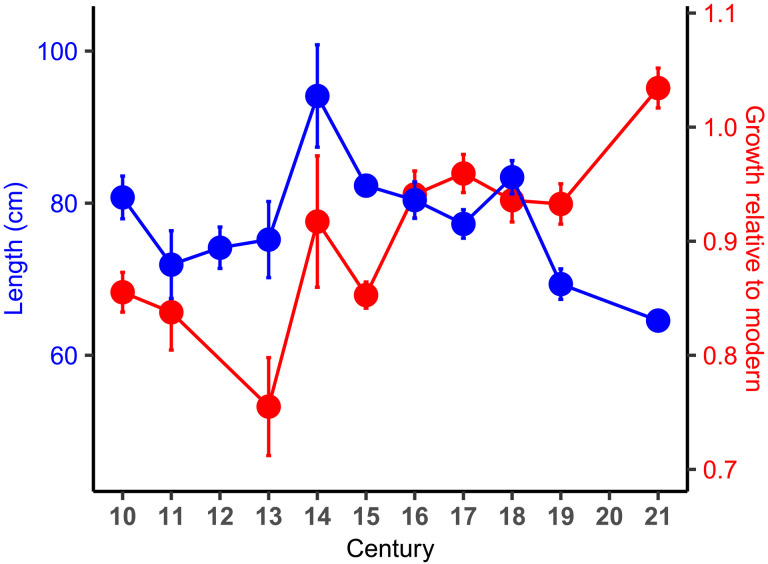
Cod length and growth rate relative to modern. Time series of cod length and growth rate [relative to modern values (years 2000 to 2010) ±1 SE] since the year 950, displayed as estimated marginal means from GLMs adjusting for regional variations. A value for the 20th century is not shown.

Estimated cod length [estimated marginal mean (EMM) from a general linear model (GLM)] declined significantly between the 10th and 21st centuries, with the largest decline taking place after the 18th century (GLM, *P* < 0.001, 872 df, *R*^2^ = 0.21) (Fig. 3). The mean length of cod in the 10th century was 80.8 ± 2.8 cm, declining to 80% of its initial value by the 21st century. The bias-corrected value (see Materials and Methods) would be expected to be 0 to 7.7% smaller than estimated for the 10th to 13th centuries, with the bias negligible for later centuries.

### Changes in age and growth across the centuries

A truncated size and age distribution is a common observation in overexploited fisheries. The 95th percentile of length did not change appreciably from 107 cm (range of 101 to 115 cm) throughout the time series. The 95th percentile of age ranged between 13 and 20 years prior to the 20th century, at which point it declined to 10 years.

Fish growth rate tends to slow after the onset of sexual maturity, making absolute growth rate sensitive to differences in age composition among samples. We used an age-invariant index of relative growth rate (*G*) based on the ratio of observed to expected length at age, which is a robust index of growth rate relative to modern cod. Interannual variation in *G* during the period 2000 to 2010 was 1.6%, demonstrating that *G* remained relatively stable over decadal timescales. *G* increased significantly by 22% between the 10th and 21st centuries, with most of the increase occurring after the 15th century (Fig. 3) (GLM, *P* < 0.001, 643 df, *R*^2^ = 0.25). Length estimation bias (see Materials and Methods) would further decrease the growth rates by 0 to 7.7% for the 10th to 13th centuries. As was the case with the time series of lengths, the largest change occurred after the 19th century. Notably, the transition period for *G* was not contemporaneous with that of length; *G* tended to increase steadily across the entire time series, whereas the length remained relatively constant until the 18th century.

### Stable isotope and temperature time series

Stable oxygen isotope ratios in otolith carbonate have often been applied to reconstruct temperature exposure histories of fish but have rarely been applied at the millennial scale. Here, they were assayed to determine whether the observed low growth rates of cod in the early centuries could be attributed to low water temperatures. Isotopic assays of modern cod from the W (3.68 ± 0.09, *n* = 6) and NW (3.97 ± 0.12, *n* = 12) regions differed only slightly [analysis of variance (ANOVA), *P* = 0.15, *n* = 18) but were in keeping with catch-weighted bottom (<200 m) water temperatures recorded in seasonal trawl surveys (W: 6.37° ± 0.04°C; NW: 5.36° ± 0.03°C). On the basis of an otolith isotope-temperature equation for cod (*35*), the isotopic differential of 0.29 between W and NW regions would correspond to an expected temperature differential of 1.38°C between regions, which was very similar to the observed temperature differential of 1.01°C.

**Fig. 4.**
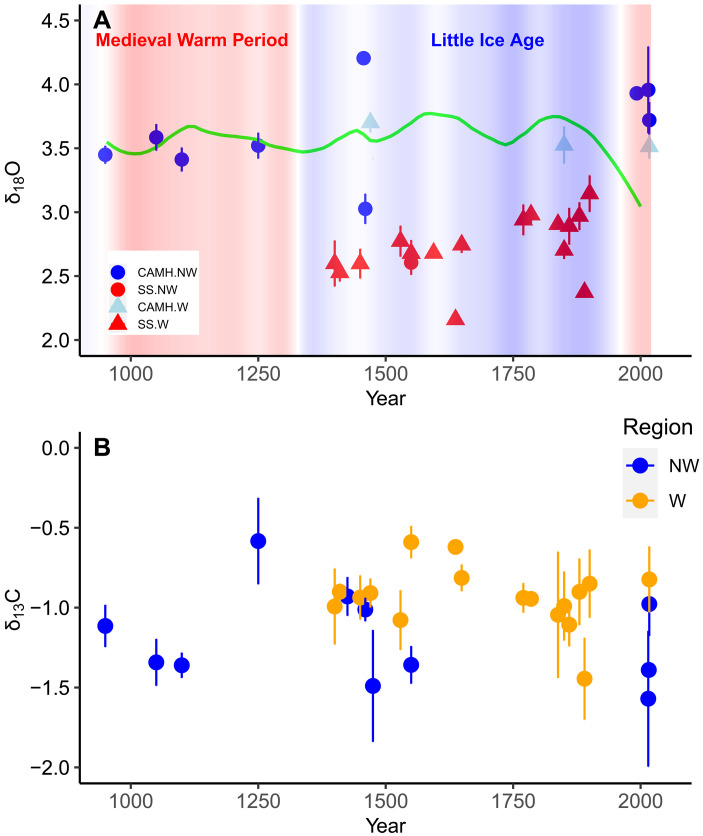
Time series in otolith stable isotopes since the year 950. (**A**) Long-term variations (± 1 SE) of otolith δ^18^O since the year 950 by dataset, region and year. There is little overlap in region or century between datasets, which complicates the interpretation of the different scaling of the two datasets. Increasing δ^18^O is known to correspond with decreasing temperatures. On the basis of oceanographic observations, the NW region tends to be cooler than the W region. Colored contours for the SST by decade off of North Iceland were drawn from Sicre *et al.* (*37*), where warm (9.2°C) is red and cool (7.1°C) is blue. The Little Ice Age between the 14th and 19th centuries is clearly evident in both the SST and the isotopic SS datasets. The green line shows the loess fit to the North Icelandic *A. islandica* δ^18^O chronology at 80 m from Reynolds *et al.* (*38*). (**B**) Long-term variations (± 1 SE) of otolith δ^13^C since the year 950 by region and year. There were no significant differences between regions or datasets nor was there evidence of a temporal trend.

Long-term trends in otolith oxygen isotopes were obtained from two independent isotopic datasets with differing coverage of region and century, which prevented intercalibration. Temporal trends within each of the two isotopic datasets were more interpretable (Fig. 4A). There was a significant increasing relationship of δ^18^O between the years of 1400 and 1900 in the SS dataset, indicative of a 1.7°C cooling trend through the Little Ice Age (*b* = 0.00072 ± 0.0002, *P* < 0.001, *R*^2^ = 0.08, *n* = 138). Although there was no significant trend between the years 950 and 2017 in the CAMH dataset (*P* = 0.2, *n* = 197), there was a significant difference in CAMH δ^18^O between the early centuries (mean of 3.46 ± 0.05 before the year 1400) and the 21st century (3.70 ± 0.09) (*P* < 0.001, *n* = 98), corresponding to a long-term cooling of 1.14°C.

Temperature reconstructions based on cod otolith carbonate do not necessarily reflect climatic trends because cod are capable of moving to preferred thermal habitats in a three-dimensional space. Nevertheless, the 1.7°C cooling trend indicated by the SS otolith isotope chronology between 1400 and 1900 was consistent with the 0.7°C cooling trend indicated by a north Icelandic sea surface temperature (SST) proxy (*37*). The 2000-year oxygen isotopic record from a north Icelandic bivalve at 80 m (*38*) (Fig. 4A) would be expected to be most reflective of cod habitat; it showed extended periods during the Little Ice Age where the δ^18^O was 0.25 greater than the preceding century (corresponding to 1.2°C cooler) but little net change either between 950 and 1900 or 1400 and 1900. The isotopic differential of about 0.5 between the years 950 and 1995 in the bivalve chronology was almost exclusively due to warming after the year 1850 and corresponded to a net warming of 2.4°C.

Stable carbon isotopes in otoliths are incorporated both from dissolved inorganic carbon and metabolic carbon and thus can be difficult to interpret (*36*). Major changes, however, can be attributed to substantive changes in the food web or in the metabolic rate of the cod population. Although both region (*P* < 0.001) and century (*P* = 0.04) were significant factors in a GLM of δ^13^C (*P* < 0.001, 325 df, *R*^2^ = 0.06), there was no significant trend across the time period 950 to 2017 (*P* > 0.2) (Fig. 4B) nor was there was a significant difference in CAMH δ^13^C between the early centuries (mean of −1.30 ± 0.06 before the year 1100) and the 21st century (−1.05 ± 0.14) (*P* = 0.09, *n* = 89).

### Cod catch and mortality rates

Mortality rate is arguably the dominating factor of any fish population. Given a known recruitment (birth) rate and a geographically isolated population, total instantaneous mortality rate (*Z*) controls the abundance, age distribution, and longevity of a fish population, with low mortality rates increasing both population abundance and longevity. The maximum observed age in the modern cod population was 16 years (*n* = 21,858), with one additional fish at age 20. The predicted longevity of 21st century cod based on the measured *Z* of 0.55 (see below) and a standard exponential mortality model was accurately predicted at 19 years. The maximum observed age of the historical otoliths between the 10th and 19th centuries, which was based on a far smaller sample size (0.2% of modern), ranged between 14 and 24 years, with maximum observed age increasing significantly with sample size (*P* < 0.05, *n* = 10). Thus, the maximum observed age of 16 years in both the 10th (*n* = 38) and 11th (*n* = 14) centuries very likely underestimated the actual longevity. In contrast, the predicted longevity using the exponential mortality model and the 10th century *Z* of 0.19 was 48 years.

**Fig. 5.**
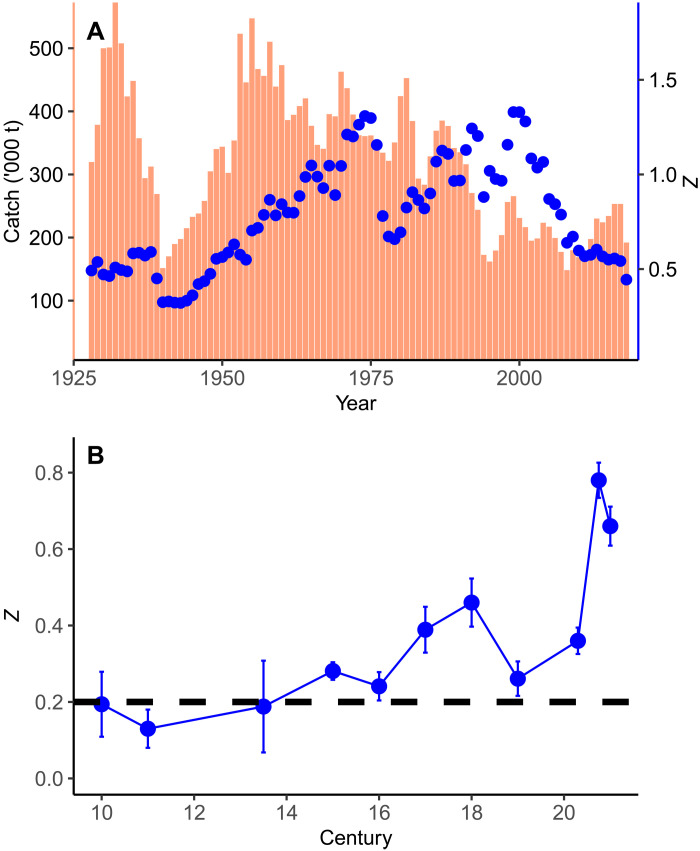
Cod mortality rate. (**A**) Annual estimates of total instantaneous mortality rate (*Z* for age 9+ fish, shown as blue points) from Icelandic cod stock assessments over the past century. Because instantaneous natural mortality is assumed to be constant at 0.2, and *Z* is the sum of both fishing and natural instantaneous mortalities, fishing mortality has been the main source of total mortality in modern times. Catches (orange bars) have been the main source of fishing mortality. (**B**) Estimates of total instantaneous mortality rate (*Z* ± 1 SE) since the 10th century indicate that *Z* was indistinguishable from the background natural mortality rate of 0.2 (dashed line) until at least the 15th century. The *Z* estimate for the 13th and 14th centuries represents a pooled estimate.

Total mortality (*Z*) in the 20th century Icelandic cod population was almost double the 0.4 threshold for optimal yield, with a mean of 0.79 (range between 0.32 and 1.33) (Fig. 5A). Fishing mortality (*F*) in the form of annual catches (ranging up to 578,000 metric tons) was responsible for most of the total mortality; the natural mortality (*M*) component of *Z* (*Z* = *M* + *F*) is extremely difficult to measure and is generally assumed to be 0.2 in cod populations (*39*). A significant correlation between catch and *Z* was both expected and observed (*P* = 0.01, *n* = 49) until the introduction of managed fishing quotas in 1977, when the correlation disappeared.

Estimates of *Z* since the 10th century indicate that it remained low until the 20th century, at which point it increased by 300% (Fig. 5B). *Z* was indistinguishable from the assumed natural mortality rate of 0.20 until at least the 14th century; *Z* in the 10th century was estimated at 0.19 ± 0.08, whereas the pooled estimate of *Z* for the 10th to 14th centuries was similar at 0.17 ± 0.09.

**Fig. 6.**
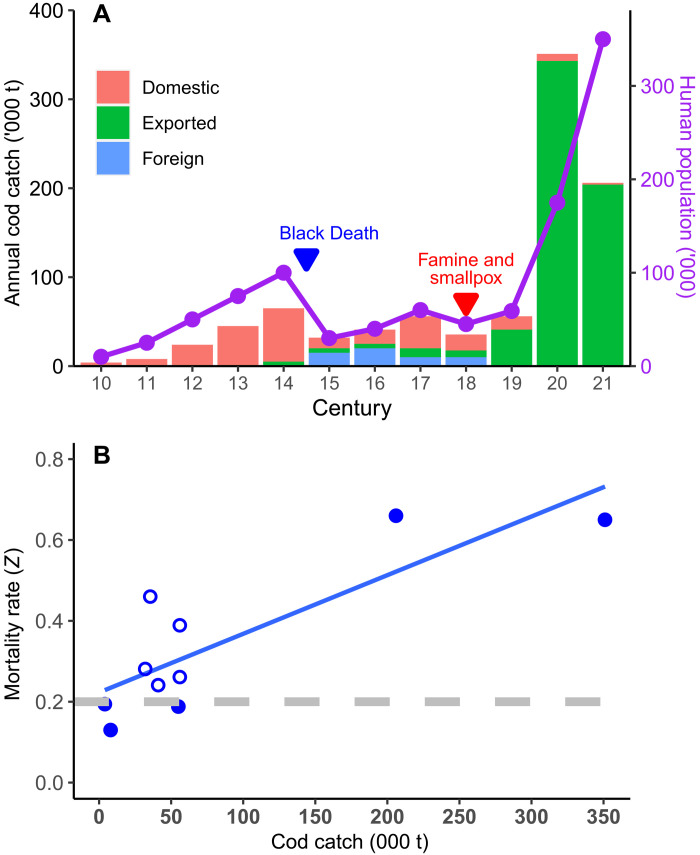
Human numbers, cod catches, and mortality since the year 950. (**A**) Estimates of annual Icelandic cod catch (‘000 t) were reconstructed from published sources and food consumption estimates for the Icelandic human population; values for the 20th and 21st century were derived from catch records. Although the domestic cod catches until the 18th century reflect the human Icelandic population fairly closely, cod catches in recent centuries have been almost completely exported. Foreign catches between the 15th and 19th centuries are suspected of being grossly underestimated. Major episodes of mass mortality (shown) resulted in substantial declines in the human population. (**B**) Annual estimates of total instantaneous mortality rate (*Z*) for each century were significantly correlated with reconstructions of mean annual cod catch since the 10th century. Filled symbols are considered robust, but the catches are probably underestimated for the centuries marked with open symbols. A linear regression (solid line) has been fitted to the relationship, although the underlying relationship is nonlinear. Dashed line indicates the mortality rate of 0.2 normally attributed solely to natural causes, not fishing. The intercept of the fitted line at *Z* = 0.19 suggests a natural mortality rate (*M*) almost identical to that commonly assumed.

Cod catch prior to the 14th century is unknown, so it was estimated based on the food consumption requirements of the human Icelandic population, which subsisted largely on cod. The estimated annual catch of Icelandic cod at the onset of fishing in the 10th century was only 4000 tons, increasing to 65,000 tons by the 14th century; the scale of these estimates is relatively insensitive to the consumption estimates upon which they are based and are believed to reflect the human Icelandic population fairly closely (Fig. 6A). Major episodes of human mass mortality in the 14th and 18th centuries resulted in substantial declines in both the human population and catches for domestic consumption. On the basis of historical records, foreign fishing first became substantial in the 15th century, but reported foreign catches between the 15th and 19th centuries are known to be incomplete. On the basis of the modern catch-*Z* relationship, the *Z* values of about 0.30 estimated for the 15th to 19th centuries (see below) would be more consistent with annual catches of 150,000 to 230,000 tons, which is five times higher than reported and estimated. The catches would be even greater if the historical cod population was more abundant than in the modern era. In contrast, modern catch estimates were reasonably well documented at a mean of 351,000 tons, almost all of which was exported.

Annual estimates of *Z* since the 10th century were significantly and positively correlated with total annual cod catch reconstructions (Fig. 6B), as would be expected if both estimates were accurate (*b* = 0.0015, *P* < 0.001, *R*^2^ = 0.84, *n* = 10). This relationship was strongly leveraged by the early (10th to 14th) and modern (20th to 21st) centuries, both of which were characterized by robust catch and *Z* estimates and which were relatively insensitive to the expected nonlinear form of the catch-*Z* relationship. The intercept of the fitted line at a catch of zero corresponds to the point when *Z* is due exclusively to natural mortality (*M*). The estimated intercept at a *Z* of 0.19 ± 0.03 was almost identical to the estimate of 0.19 ± 0.08 previously estimated for the 10th century.

### Relationship between growth rate and abundance

**Fig. 7.**
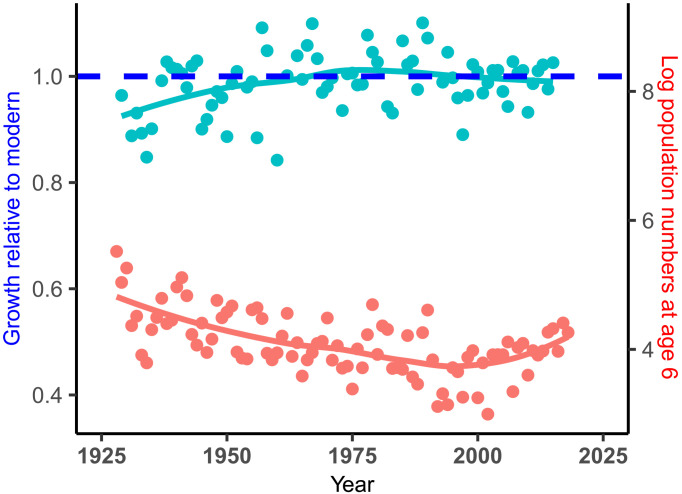
Relationship between growth rate and abundance over the past century. Cod growth rate (relative to modern, shown as dashed line) at age 9 varied inversely with adult population abundance (as indexed by age 6 numbers) over the past century, a period of intense fishing and relatively low abundance.

The annual growth rate and population abundance of Icelandic cod were both known with considerable accuracy after 1925, thus allowing their interrelationship to be assessed at the annual timescale during a period of intense fishing and low abundance. Relative growth rate (*G*) increased through the 20th century, reaching an asymptote around the year 1955 (Fig. 7). The population abundance of adult cod (as indexed by age 6 numbers) declined through this same period, although the correlation was not significant (*P* > 0.1). However, the cross-correlation was significant when abundance was lagged by 6 years (*r* = 0.4, *P* < 0.01, *n* = 76), implying that the growth index responded relatively quickly to changes in abundance.

**Fig. 8.**
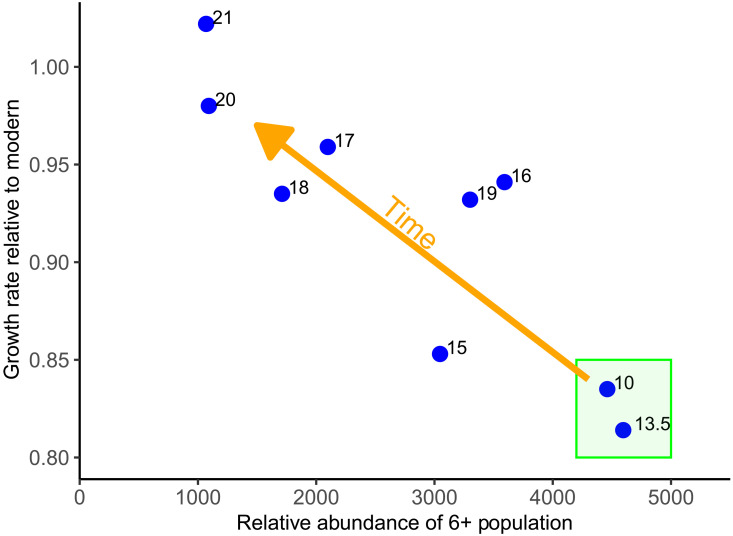
Relationship between growth rate and abundance since the year 950. Cod growth rate (relative to modern) increased significantly as estimated population abundance decreased; note that time proceeds from right to left in the plot. Point labels indicate the century. The green box indicates the centuries where there has been no apparent impact of fishing (either catch or mortality) on growth rate. The period after the 14th century represents a transition point from a region of apparent stability in growth rate and population abundance/density (green box) to a more exploited state with relatively rapid growth as density-dependent effects disappear.

Density-dependent growth would be expected to be maximal in an unfished population, when abundance is at its peak, and then decline as population numbers decline with fishing. Estimated adult cod abundance declined almost linearly after the 10th century, with the notable exception of the 15th century (Fig. 8). Adult abundance was estimated to be a factor of 5.7 larger in the 10th century than the mean of the 20th century and 21 times larger than the minimum observed modern annual adult cod population estimate (in 2002). Relative growth rate (*G*) increased by 20% over the same period. There was a significant inverse relationship between *G* and estimated population abundance over the 1100-year time series (Fig. 8) (*P* < 0.001, 9 df, *R*^2^ = 0.71). The period around the 15th century appeared to represent a transition point from an era of apparent stability in growth rate and population abundance/density (10th to 13th centuries) to a more exploited state with relatively rapid growth as density-dependent effects disappeared.

Although population abundance explained a significant amount of the variance in *G* and was highly correlated with *Z* (r = −0.94, *P* < 0.001, 11 df), other possible growth modifiers include *Z*, temperature, and food supply. *Z* was significantly correlated with *G* (*r* = 0.80, *P* < 0.01, 11 df), but temperature (otolith δ^18^O; *r* = 0.53, *P* = 0.11, 8 df) (bivalve δ^18^O; *r* = −0.44, *P* = 0.10, 13 df) and food (using δ^13^C as a proxy) (*r* = 0.002, *P* > 0.1, 8 df) were not. A hierarchical GLM of *G* against adult abundance, *Z*, δ^18^O, and δ^13^C left only adult abundance as a significant factor (*P* < 0.01, 7 df, *R*^2^ = 0.75). Therefore, adult cod abundance appeared to be the primary explanation for relatively slow growth through the early centuries.

## DISCUSSION

The fortuitous combination of recent human colonization (*40*), the precise dating of postsettlement history using radiocarbon, detailed Icelandic sagas and volcanic tephra layers (*41*), and the geographic isolation of one of the world’s largest and most productive cod stocks (*42*), has allowed a rare insight into the population-level response of an untouched cod population to 1100 years of increasingly intense fishing. On the basis of the size of the human Icelandic population and the absence of a long-distance fishing fleet shortly after settlement, the 10th to 12th centuries represent a period during which fishing effects on the large pristine fish population were negligible. Our results indicate that an initial abundance that was 570% greater than modern was only one aspect of a multifaceted shift in cod population dynamics after the onset of fishing. Cod during the early centuries grew to an age that was almost three times older than modern and were caught at a length that was 25% larger, despite the use of less-developed fishing gear and a growth rate that was 22% slower than modern cod. Perhaps unexpectedly, their natural mortality rate remained at the same level as is now assumed for the modern population; due to the absence of any appreciable fishing mortality, total mortality in the early centuries was just 24% of that of modern cod.

A substantial decline in prey abundance due to harvest of standing biomass is a predictable result of the onset of human exploitation of a human food source. Although extinction-level changes in the abundance of Pleistocene era herbivores may be an extreme example of human harvesting (*43*), abundance declines exceeding 90% appear to characterize the early stages of fishing on several marine predatory fish populations (*44*). Comparative studies of fished and unfished reef fish populations have reported a 260% difference in predator abundance and biomass (*45*), whereas 5 to 21% reductions in size and age at maturity and spawning have been reported for a broad suite of global fish populations after exploitation (*46*). The key demographic rates of growth, mortality, and recruitment that govern the abundance of all populations is virtually unknown for unexploited fish populations due to the absence of fisheries monitoring data prior to the past century (*47*). Although demographic studies of recovering populations have all reported changes in size and age structure with increasing abundance, increasing and decreasing population trajectories are often not equivalent; ecosystem changes over time, Allee effects, and regime shifts in the recovering population can all cause marked differences from the original population structure and abundance (*47*–*49*). Thus, it is remarkable that the population characteristics of the 10th century Icelandic cod population were not even more distinct from those of the 21st century. Given the exponential increase in ovary size and egg production with increasing female length, the presence of so many large and old fish in the unfished population probably increased egg production well beyond that expected of the predicted high adult abundance (*7*). Although we were not able to estimate historical recruitment or age at maturity in this study, there is no evidence of depensatory recruitment in Icelandic cod (*42*) and thus no evidence that recruitment was any less than it has been in the past century. The opposite appears likely, which would have inflated our historical abundance estimates to even higher levels. Given that the marine circulation around Iceland is both energetic and highly productive (*50*), cod abundance in the Viking era may have been limited more by available habitat than by any other single constraining factor on carrying capacity.

The instantaneous rate of natural mortality (*M*) is notoriously difficult to estimate in exploited marine populations because it can be confounded by immigrants from neighboring populations and with co-occurring fishing mortality (*F*) (*51*). In this study, the very low level of fishing in early centuries and the relative isolation of the Icelandic cod population allowed a more robust estimation of *M*. The assumed, fixed value of 0.2 for *M* used in modern groundfish population models has achieved almost mythical status, with its origin often attributed to a mistakenly transcribed “?” (*52*). Recent studies have demonstrated that *M* is not necessarily stable across the entire spectrum of population abundances, with lower than expected values observed in pristine lake trout populations (*8*) and higher than expected values observed in heavily exploited cod populations undergoing a major ecosystem regime shift (*53*). Our findings highlight a remarkable stability in the natural mortality rate of cod in an unexploited population in an unperturbed ecosystem 1100 years ago (0.17 to 0.19) relative to the 0.20 value now assumed for the modern fishery, a finding that has implications for conservation population modeling in other species.

A low total mortality rate, resulting from the absence of any detectable component due to fishing, appears to have been responsible for most or all of the characteristics of the pristine cod population prior to the 15th century. Without the need to invoke additional causes, the progressive increase in *Z* across the centuries can fully account for the reduced population abundance, reduced longevity, disappearance of older age classes, and decline in mean length, all of which are commonly observed symptoms of an overexploited population (*54*). Increasing mortality and its accompanying production of a lower population density could also account for the observed 20% increase in growth rate through time because density-dependent growth has often been documented in fish populations (*6*, *55*). Centennial changes in water temperature and the potential for altered food webs and regime shifts (*56*) must have been present, but it appears that ever-increasing levels of exploitation alone, caused by increasing Icelandic and foreign fishing capacity, dominated the 1100-year trajectory of the Icelandic cod population. A similar conclusion was reached in a zooarchaeological study of Scandinavian cod wherein stabilizing “supergenes” appeared to promote population stability in the face of environmental challenges but not increasing fishing pressure (*57*).

Density dependence in fish and other poikilotherms can be very different from that of mammals due to the fish’s indeterminate growth trajectory and the dominant influence of temperature on fish physiology (*58*). Density dependence would be expected to be maximal in an unfished population, its effect declining with population abundance. Yet, it is rare to obtain a time series where the full range of density-dependent responses is on display. Density-dependent growth variations of 10% in the 20th century were only one-half of those apparent over the entire 1100-year time series, despite a 25-fold variation in annual catch and population abundance since 1900. Nevertheless, our results suggest that the density-dependent process was time invariant—growth variations in the 20th century were muted relative to those prior to the 15th century solely because the original abundance was so much greater than that in modern times. Several possible alternatives to density-dependent growth were not supported by our results; there was no evidence of long-term increases in water temperature, which could have elevated growth rates, nor was there any evidence in the carbon isotopic signature signifying major shifts in the historical ecosystem. Temperature and food are well-known modifiers of fish growth so undoubtedly would have modified cod growth at the annual timescale. However, interannual fluctuations in temperature and food supply are almost imperceptible at the century scale, lending credence to the proposition that reduced density dependence alone increased growth rate through the time series. Similar density-dependent growth responses were reported for a porbeagle shark (*Lamna nasus*) population before and after the onset of fishing (*59*).

Fisheries-induced evolution is believed to favor more rapid maturation and slower postmaturation growth (*60*) but was not detected in our study. Genetic heritability studies increasingly support the premise that fisheries-induced evolution can select for changes in life history traits, appearance, and behavior (*61*). There is also evidence that population productivity (*r*) can be genetically altered at high harvest rates (*62*). However, the long-term increase in adult growth rate observed in our study is not consistent with fisheries-induced evolution of growth rates nor is it consistent with the rapid response of growth rate to changes in population abundance in the past century. Our conclusions are supported by recent analyses suggesting that evolutionary changes in growth rate should be slow and of much smaller magnitude than the phenotypic manifestations of high mortality and depleted population abundance (*61*, *63*).

The increasing trajectory of inferred cod catches did not appear to affect the population dynamics until the 14th century, when a relaxation of density dependent growth first became evident. The first appearance of an elevated total mortality rate in the 15th century would appear to be at odds with the simultaneous and abrupt decline in catches, but it is the catches which are almost certainly underestimated. Icelandic historical writings refer to the 15th century as the “English century,” a period during which 150 ships were dispatched annually from England to fish for cod in Icelandic waters (*19*). The number of English ships declined steadily beginning in the 16th century, coincident with their appearance off Newfoundland, Canada (*14*). A translation of a letter written by a diplomat in London in 1497 reported that “…This sea (by Newfoundland) is so full of fish that it not only is caught in nets but also with a basket... They say that so much of fish will be transported from there that this kingdom (England) will have no more need for Iceland...” (*64*). The 16th century annual Newfoundland catches of 140,000 tons (*14*, *27*) were almost certainly a translocation of the Icelandic fishery and were very similar to our estimate of 150,000 tons in 15th century Iceland based on the calculated fishing mortality rate. Incomplete historical records of fish catches are difficult to recognize, but it appears that fishing mortality rates based on otolith age compositions can provide a quantitative estimate of past catches as long as the catch-*Z* relationship is known. In principle, the use of *Z* for estimating the magnitude of unrecorded catches can even be adjusted for gear selectivity.

Pinnegar and Engelhard (*13*) concluded that “ecosystems were not pristine before the onset of industrial fishing and it is difficult to assess the ‘virgin’ state of a population given that it may have been subject to moderate or even high levels of fishing mortality for many centuries,” an assessment that was justifiably echoed by Holm *et al.* (*14*). However, it may be time to revisit that conclusion. Modern fish stock assessments, population models, and conservation recovery reports are primarily based on age, growth, and mortality estimates derived from otoliths (*65*), so it is reasonable that archaeologically excavated otoliths could be used to reconstruct most aspects of the age-structured dynamics of historical fish populations. Of course, application of *Z* to estimate relative abundance assumes a stable stock-recruitment relationship (*42*), which may not exist if age at sexual maturity varies with population density (*66*). An alternative metric for abundance or population density could be the relative growth index *G* used in this study. *G* was observed to vary with density at the annual scale in the 20th century, which suggests that it responded quickly to changes in abundance. If properly controlled for environmental effects in a mixed effects model (*6*, *67*), *G* could conceivably be used as a biological reference point and an index of modern population abundance.

## MATERIALS AND METHODS

### Archaeological collections

The focus of this paper is on otoliths from Atlantic cod (*G. morhua*) sourced from Icelandic archaeological sites. Only sites with well-preserved otoliths identifiable to the species level were included. Our study sites were all involved in the processing of gadids for domestic consumption or regional trade and export. The processing of gadids through air drying them into a variety of dried fish products dates to the first settlement of Iceland and was likely a technology brought to Iceland by the first Norse settlers (*17*, *30*).

All archaeological sites were excavated following the protocol established by the Institute for Archaeology, Iceland (Fornleifastofnun Íslands), which is a modified single-context excavation protocol based on Museum of London Archaeological Service procedures. All excavated deposits were dry-sieved through 4 mm mesh.

Faunal materials were analyzed in accordance with standardized North Atlantic Biocultural Organization (NABO) methodologies using the ninth edition of the NABONE recording package, consisting of a Microsoft Access database and specialized Microsoft Excel spreadsheets. The recording package is available for download at www.nabohome.org. Analyses were performed at the zooarchaeology labs at the University of Maryland, the Hunter College (CUNY), and the University of Iceland labs at Reykjavik and the Research Center in the Westfjords Region.

The dating of the materials depended on the specific conditions of each site. Dating techniques included radiocarbon dating, tephrochronology, and, for early modern sites, dating through artifacts.

The identification of cod otoliths followed the recommendations of the International Council for Archaeozoology (ICAZ) Fish Remains Working Group. Species identification was confirmed at the Campana Lab at the University of Iceland based on sagittal otolith morphology, which is distinctive for gadiform species (*68*). Of 613 otoliths, 92% were confirmed as being from Atlantic cod (*G. morhua*) and 0.3% from haddock (*Melanogrammus aeglefinus*), whereas 7.6% were definitely gadids and presumed to be cod. Haddock otoliths were not used in this study. Otoliths showing evidence of dissolution or loss of the margins were clearly identifiable and were not used.

### Modern cod

The otolith characteristics of the historical cod were compared with two independent sources of modern cod from the study area: (i) annual standardized spring and autumn groundfish trawl surveys conducted by the Marine and Freshwater Research Institute (MFRI) and (ii) MFRI samples of the commercial hook and line fishery. Only cod collected in the years 2000 to 2010 in the waters between latitudes 64.2°N and 67°N and between longitudes of 18.55°W and 26°W were used. Research vessel trawls were fitted with small mesh designed to catch all fish sizes, and thus the data were truncated at the minimum observed length in the commercial hook and line fishery (32 cm). All analyses of modern length composition were restricted to the hook and line fishery, which most closely matched the hook and lines used in the historical fishery. The remaining analyses were based only on the research survey samples, which best reflected the actual length composition in the study area. However, the length compositions of the research survey and hook and line fishery were similar for adult fish (fig. S3), and none of the results were sensitive to the dataset that was used. For comparison of stable isotopes, adult cod otoliths (*N* = 18) were collected in the years 2015 to 2017 from Skagafjörður (66°N 19.5°W to 20.0°W), Breiðafjörður (65.3°N 23.8°W), and Ísafjarðardjúp (66.3°N 23.3°W).

### Otolith preparation

A key advantage of the age-based dynamics reported in this study was the use of carefully calibrated fish age determinations using modern embedding, sectioning, and image analysis methods of demonstrated accuracy and precision (*67*, *69*). Ages were based on counts of annual growth increments that were visible under reflected light in transverse sections of the sagittal otolith, and all otoliths were processed identically and aged by the same expert age reader [coefficient of variation (CV) < 3.8%]. The same age reader was involved with the age interpretations of the modern cod by MFRI. Thus the age, growth, and mortality estimates for all fish and time periods are completely comparable.

Adult cod otoliths increase in size as a function of fish growth, thus enabling estimation of fish length based on otolith dimensions (fig. S2). The relationship between cod length and the diameter of the otolith transverse section along the dorsoventral axis was determined using 114 modern cod otoliths of a broad size range from the study area, resulting in the following predictive relationship

$$\text{Ln}\;F=1.366\;\left(\text{Ln}\;O\right)-8.05\;\left(P<0.001,R^{2}=0.95\right)$$

where *F* is fish length in cm and *O* is otolith width in μm. Otolith size in slow-growing fish is known to be slightly greater than that of fast-growing fish of comparable length (*70*). Therefore, use of a modern, fast-growing fish-otolith relationship to reconstruct fish length from slow-growing historic otoliths should result in fish length and growth rate estimates that are slightly larger than actual, an effect that was confirmed in an analysis of our modern fish-otolith data. The extent of the bias was estimated at 7.7% length overestimation given a 20% reduction in fish growth rate (corresponding to the lowest observed), with the bias approaching zero as relative growth rates increase.

### Growth, longevity, and mortality estimation

The growth rate of a population of fish over its lifetime can be modeled as a function of fish length and age, but ontogenetic and regional changes in growth rate complicate any comparison of growth rates from samples with different age compositions. To calculate an age-independent index of relative growth rate, a von Bertalanffy growth model was first fit to our large sample of modern cod from the study area (*N* = 14,885), where

$$L_{t}=L_{\infty }\left[1-e^{K\left(t-t_{0}\right)}\right]$$

The resulting model produced estimates for $L_{t}$ (the total length of the fish in cm at age t in years), $L_{\infty }$ (the asymptotic length = 148.0 cm), *K* (a growth coefficient = 0.098 years^−1^), and $t_{0}$ (the age at zero length = −0.63). This model fit served as a modern baseline against which all other length at age measurements could be compared. Thus, the ratio of the observed length at age in fish *i* relative to the modeled length at age *a* is an index of growth rate (*G_ia_*) relative to a modern cod of the same age, with values less than 1 indicating slower growth and values greater than 1 indicating more rapid growth. Because *G* is a proportional index, it is age independent and can be compared with estimates of *G* from fish with different ages with no change in interpretation. Age and size independence was confirmed in analyses of the modern cod from different areas. *G* was then calculated for all of the modern and historical cod where length and age estimates existed. *G* estimates used for the comparison with population abundance for the period 1928 to 2015 were derived from observations of length at age 9 cod (*39*).

Total instantaneous mortality rate (*Z*) was estimated from age composition data using two approaches: traditional catch curve (CC) and the Chapman-Robson (CR) estimator. Although the CR estimator has been shown to be superior to most other traditional catch-curve analyses for small sample sizes (*71*), it assumes that old fish are fully recruited to the fishing gear. Some of our historical samples showed evidence of truncation at large sizes, thus artifactually increasing the CR estimate of *Z* but leaving the CC estimate unaffected. Therefore, estimates of *Z* were calculated using both methods, and the mean value (weighted by the inverse of the SE) used in subsequent analyses. Minimum sample size for *Z* estimation was set at 10, which would have excluded the 14th century, so it was calculated as a pooled estimate with the 13th century. The sample size was sufficiently large for the 10th century that mortality estimates were generated for each of the three date contexts and then averaged. There were insufficient ages for a 12th century mortality estimation. The minimum age for *Z* estimation was set at age 9, unless the age composition clearly peaked at an older age and sample sizes were sufficient. A comparison of *Z* between the weighted mean *Z* estimate from modern cod and the *Z* from reported stock assessment models indicated close agreement (±9%), so the latter was used for the 20th and 21st centuries. The fully recruited age composition and *Z* estimates from the modern hook and line fishery were comparable to those of the research vessel surveys from the same area and time period (fig. S4). In an unfished population, *Z* is equal to the instantaneous rate of natural mortality (*M*).

The observed maximum age is a valid estimate of longevity in an unfished population, but increases asymptotically with sample size. If *Z* is known, an alternative approach is to predict the surviving numbers at fully recruited ages using a standard exponential mortality model (see the section Cod catch and abundance chronology). We used the number of cod at age 6 (= 3195) in the modern cod sample as a starting value and defined longevity as the age at which the number of predicted survivors reached 1. This approach correctly predicted the maximum observed age in the modern sample.

### Stable isotopes

Stable isotopes of carbon and oxygen were extracted from both archaeological and modern otoliths to search for temporal changes in either metabolic/dietary (δ^13^C) or thermal (δ^18^O) history. Otolith isotopes were assayed using the general approach described in von Leesen *et al.* (*72*). In brief, otolith samples for assay were first extracted from the transverse sections used for age determination with a computerized micromilling machine. Approximately one-third of the otoliths (SS dataset) were micromilled at two discrete locations in the section: the region encompassing the first year of life (juvenile) and again during the fifth to seventh year of life (adult). Assays of the remaining archaeological otoliths (CAMH dataset) used all or part of the section (across ages) due to breakage. All samples were then reacted with dehydrated phosphoric acid under a vacuum at 70°C to produce CO_2_. The SS samples were analyzed for δ^13^C and δ^18^O using an automated carbonate preparation device (KIEL-III) coupled to a gas-ratio mass spectrometer (Finnegan MAT 252) at the University of Arizona with an analytical precision of ±0.1‰ for oxygen and 0.08‰ for carbon. The CAMH samples were analyzed at the Alaska Stable Isotope Facility (ASIF) at the Water and Environmental Research Center at the University of Alaska Fairbanks. Stable isotope data were obtained using continuous-flow isotope ratio mass spectrometry (CFIRMS). δ^13^C and δ^18^O values were measured using a Thermo Fisher Scientific GasBench II carbonate analyzer with a Thermo Fisher Scientific DeltaV^Plus^ Mass Spectrometer with an instrumental precision <0.2 ‰ for carbon and <0.4‰ for oxygen. Stable isotope ratios were reported in δ notation as parts per thousand (‰) deviation from the international standards Peedee Belemnite (PDB).

To calibrate the age-specific and age-aggregated strategies, mean δ^13^C values were compared in otoliths matched to region (W) and century (15th and 19th) in a matched pair design (*N* = 125). The resulting comparison indicated that a weighting of 30% juvenile and 70% adult produced a mean δ^13^C value for SS otoliths similar to that of the whole-section CAMH assay. The same weighting was then applied to the age-structured δ^18^O values to produce a single value per otolith.

Modern otoliths were powdered prior to assay and not sectioned. δ^13^C values from these otoliths were consistent with the sectioned adult δ^13^C time series, as would be expected of an intact adult otolith aged 6 to 12 years. To calibrate the modern whole-otolith assay values with the remaining age-aggregated section assay values, the mean within-section difference between the adult and age-weighted section δ^13^C values was calculated (= 0.44) and then subtracted from the modern whole-otolith assay values. The corresponding difference and calibration for δ^18^O was 0.17. Modern δ^18^O values were first corrected for Suess effects (*73*). The net result was a δ^13^C time series, which was fully comparable across preparation methods, datasets, and centuries. For reasons possibly related to differing water mass composition across centuries, the δ^18^O chronologies were consistent only within datasets. Therefore, each dataset was examined separately.

There are no long-term time series of water temperature at depth for the study area. Our time series of the SST for northern Iceland was based on a spline fitted to a 2000-year SST chronology (*37*). A 1000-year chronology of aragonitic *Arctica islandica* shell δ^18^O values was available from an 80-m depth in North Iceland (*38*).

### Cod catch and abundance chronology

The stock dynamics of Icelandic cod is well documented for the period after 1955 (*74*) and somewhat less so for the early years (*75*). Development of a single consistent time series of catch, biomass, recruitment, and mortality extending back to 1928 was described by Campana *et al.* (*39*). However, stock assessments are not available for earlier periods, and even the catch history is absent or incomplete for many centuries. Therefore, a complete catch history dating back to the initial settlement of Iceland in the year 874 was reconstructed based on several sources.

Historical catches of Icelandic cod fall into three categories: fishing by foreign fleets, exports from Iceland, and consumption within Iceland. Cod catches by the English, Dutch, and French fleets after the 16th century have been estimated (*24*) but were reported as being incomplete (*27*). Cod exports in the 17th and 18th centuries have also been estimated (*24*, *25*), as have catches by the Icelandic fleet (*24*–*26*). The cod consumption within Iceland was calculated based on the assumed human population size and the knowledge that much of the food energy for Icelanders necessarily came from fish and primarily from cod. The following consumption rate per capita per period was assumed: 400 kg for the years 877 to 1200, 600 kg for 1200 to 1400, 400 kg for 1400 to 1600, 600 kg for 1600 to 1700, and 400 kg for 1700 to 1800. The size of the Icelandic population between 877 and 1800 was reported here and elsewhere (*76*).

A cod population abundance time series for the period 1928 to 2018 has been published (*39*), but standard population modeling approaches could not be applied to earlier centuries. However, the age-based total instantaneous mortality rate estimates described above incorporate all sources of mortality (natural and fishing) for fully recruited fish ages. Therefore, given an assumed annual recruitment level (as is used for modern catch projections), it is straightforward to predict numbers at age using a standard exponential mortality model, as in

$$N_{1}={N_{0}}^{*}e^{-\mathrm{Zt}}$$

where *N*_0_ is the number at the age of recruitment, *N*_1_ is the number at time *t*, and *Z* is the total instantaneous mortality rate (year^−1^). The calculated numbers at age were summed across ages 6 to 21 to produce an expected adult abundance index for each century. Temporal changes in partial recruitment would affect the estimated population time series but only if fishing mortality was high (which it was not). Higher recruitment in early centuries due to high spawning stock biomass would act to increase early apparent abundance not decrease it. Thus, the mortality-based population abundance time series should provide a relative abundance time series for adult cod, which is consistent across premodern centuries. For the modern period (1928 to 2018), the time series for Z was further subdivided into partial centuries (1928 to 1975; 1975 to 1999; 2000 to 2018).

### Data preparation and statistical analysis

After exploratory analysis, individual otolith collections were aggregated at the century and regional level. Analysis of the modern data indicated that cod growth differs significantly between the cool northern waters of the Icelandic shelf and the warmer western and southern waters. Therefore, all linear models of cod length, growth, and isotopic composition included region as a factor in the analysis, with the NW region defined as the waters west of 18.5° and north of 66° (north of 65.4° when east of 22°), whereas the W region was defined as the waters between latitudes 64.2° and 66° and west of 22°. For the modern cod (20th and 21st centuries), the westward extent of the data was truncated at a longitude of 26° and the northward extent bounded by a latitude of 67°. Marginal mean parameter estimates for century, which controlled for regional differences, were used in all time series plots. Regional differences were not evident in the mortality estimates so were not used there.

GLMs were fit with the lm function in *R* (*77*), whereas expected marginal means were generated with the emmeans function (*78*). Model selection was based on the Akaike information criterion (AIC) as implemented in the R library MASS (*79*). The von Bertalanffy growth model was fit in the R package fishmethods (*80*), whereas the mortality rates were estimated in the package fsa (*81*).

All means are reported ±1 SE.
