# Supplementary material for: Mortality drives production dynamics of Atlantic cod through 1100 years of commercial fishing
Source: Sci Adv. 2025 Feb 5;11(6):eadt4782. doi: 10.1126/sciadv.adt4782 (PMC11797545; doi:10.1126/sciadv.adt4782)
Supplement: Supplementary file 1 — Figs. S1 to S4 Table S1 [file sciadv.adt4782_sm.pdf]

Science Advances
AAAS

Supplementary Materials for

Mortality drives production dynamics of Atlantic cod through 1100 years of commercial fishing

Steven E. Campana *et al.*

Corresponding author: Steven E. Campana, scampana@hi.is

Sci. Adv. **11**, eadt4782 (2025)
DOI: 10.1126/sciadv.adt4782

This PDF file includes:

Figs. S1 to S4
Table S1

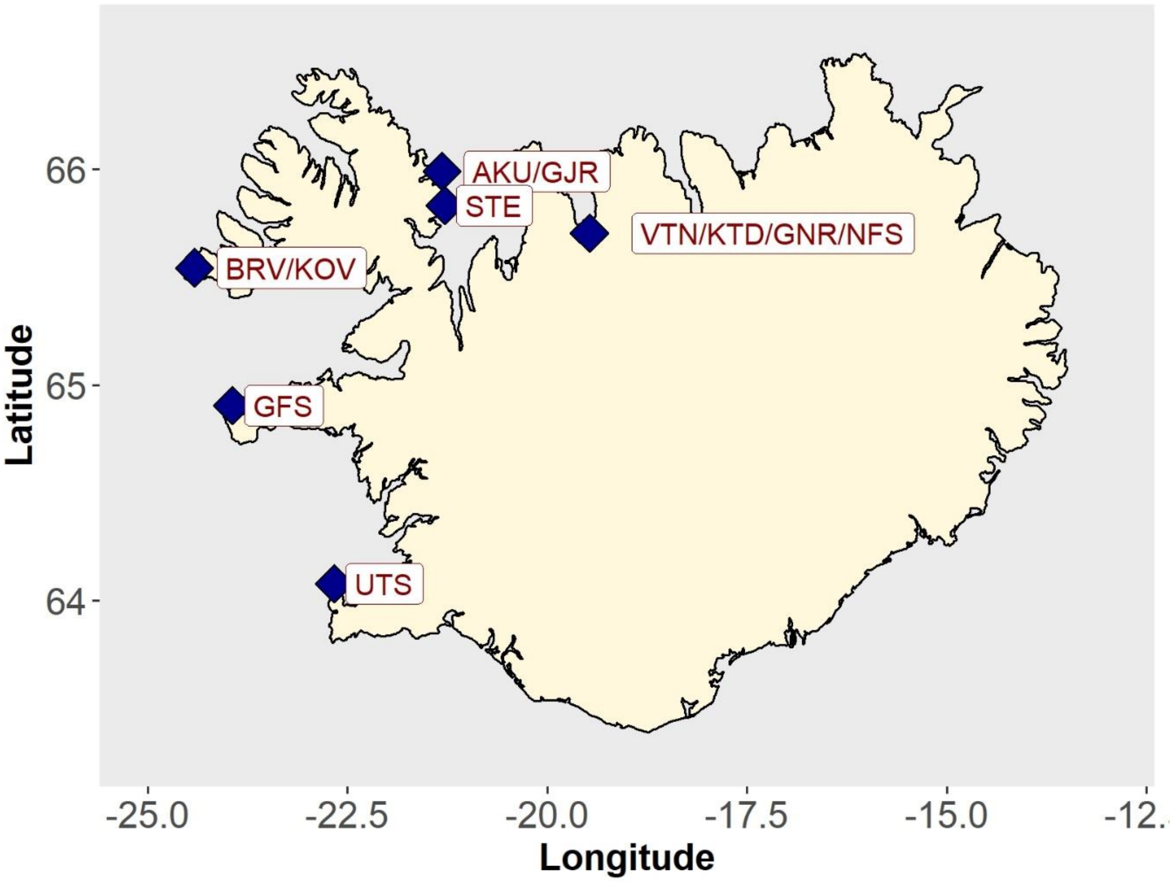

Fig. S1. Map of study area. Site details shown in Table S1.

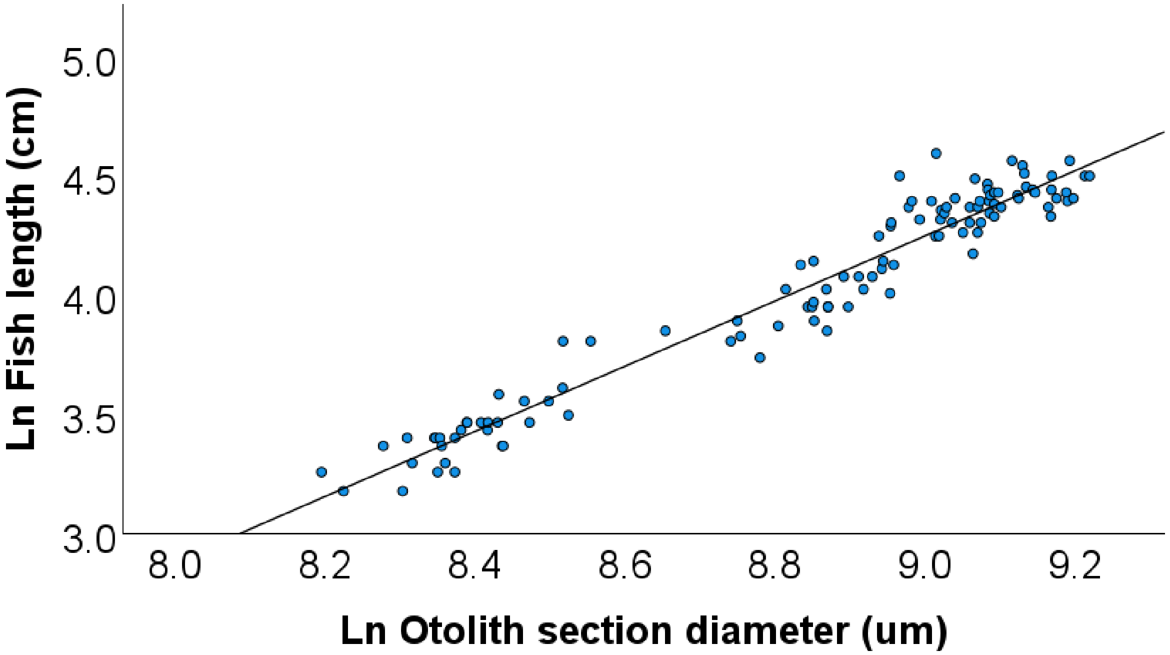

Fig. S2. Relationship between fish length and otolith section diameter used to reconstruct the lengths of the historical cod. Otolith sections were prepared from cod of known length (n=114) that were collected from research surveys of the study area in 2018. Ln FL = 1.366 * Ln Oto – 8.049; $P<0.001$, $R^2=0.95$.

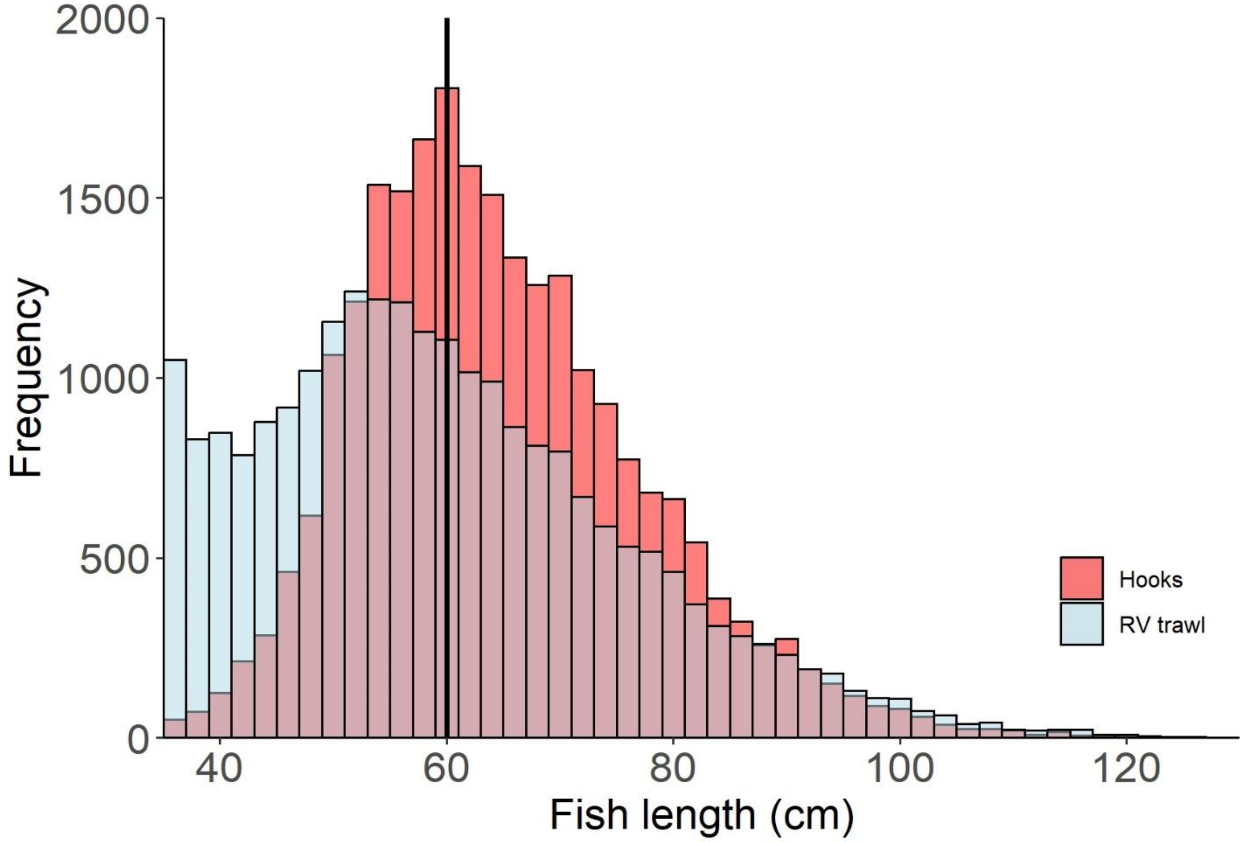

Fig. S3. Length composition of modern cod. Cod lengths recorded in spring and fall research vessel surveys (blue) and the commercial hook and line fishery (red) conducted between the years 2000-2010 in the study area. Research vessel trawls are fitted with small mesh designed to catch all fish sizes, and thus were truncated at the minimum observed length in the commercial hook and line fishery (32 cm). The mean length of cod caught in the trawl survey was 59.4 cm (n=23,981), while that in the contemporaneous hook and line fishery was 65.1 cm (n=24,324) Solid vertical line indicates the modal length of cod (= 60 cm) in the hook and line fishery, which is 6 cm larger than that in the research vessel surveys.

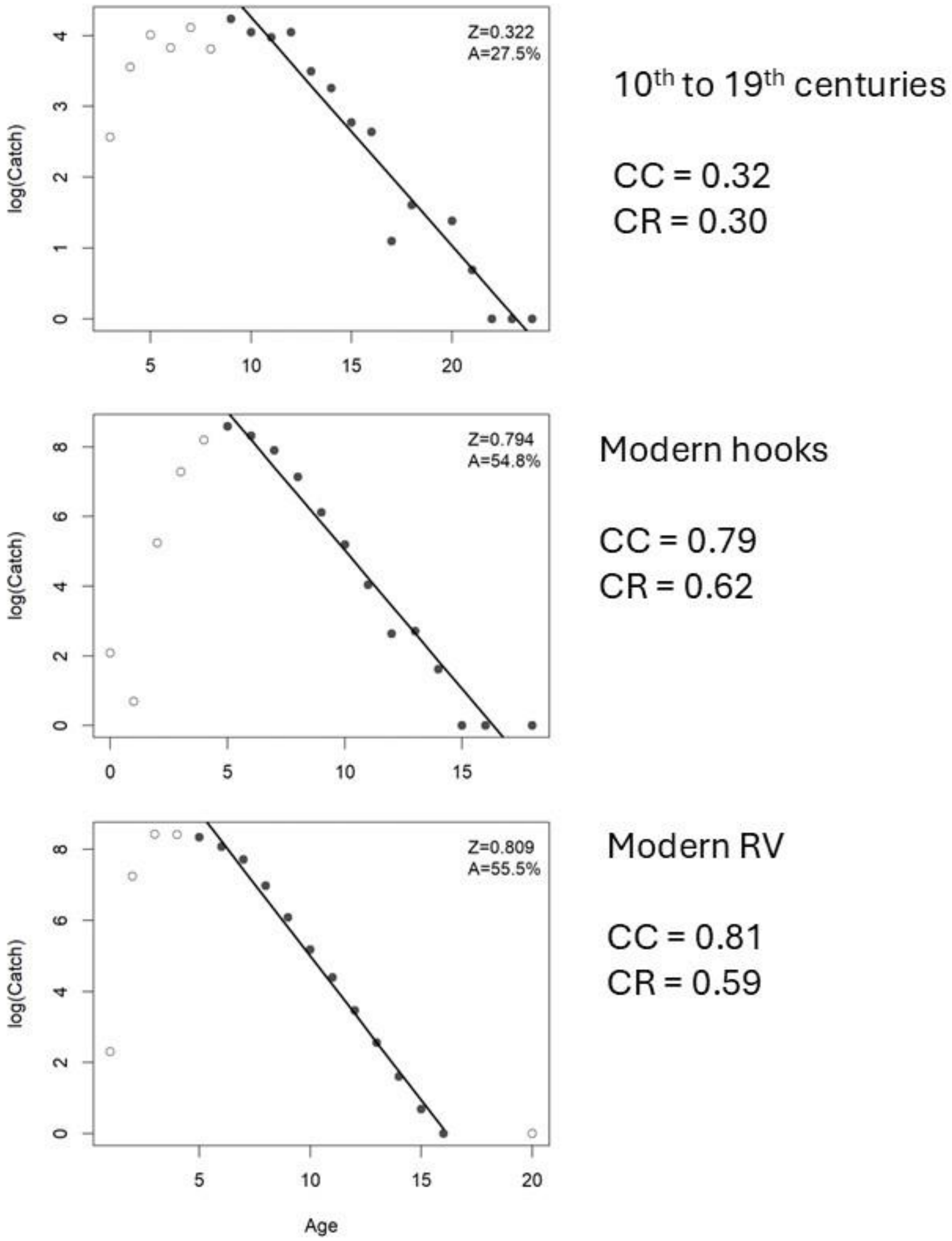

Fig. S4. Age composition of historical (10th to 19th centuries combined), modern hook and line (2000 to 2010) and modern research vessel (RV) survey (2000 to 2010) cod from the study area. Mortality estimation was based on the mean of catch curves (CC) and the Chapman-Robson method (CR) from fully recruited fish (solid symbols). Open symbols show fish ages not used in the mortality calculation.

Table S1. Sample site information.

Site	SiteCode	Region	Year	Century	N lengths	N ages	N isotopes
Akurvík	AK22	NW	1460	15	31	34	34
Akurvik	AK30	NW	1250	13	11	12	12
Akurvik	AKU	NW	1475	15	2	2	2
Breiðavík	BRV-2012	W	1554	16	0	3	0
Breiðavík	BRV-2012	W	1621	17	7	10	0
Breiðavík	BRV-2012	W	1850	19	0	2	0
Breiðavík	BRV12	W	1594	16	1	1	0
Breiðavík	BRV12	W	1850	19	2	2	2
Breiðavík	BRV15	W	1637	17	1	1	1
Breiðavík	BRV15	W	1649	17	1	1	1
Breiðavík	BRV15-1	W	1820	19	10	10	0
Breiðavík	BRV15-12	W	1812	19	9	9	0
Breiðavík	BRV15-13	W	1649	17	9	9	0
Breiðavík	BRV15-14	W	1550	16	10	10	0
Breiðavík	BRV15-15	W	1550	16	10	10	0
Breiðavík	BRV15-16	W	1410	15	45	45	0
Breiðavík	BRV15-17	W	1410	15	5	5	0
Breiðavík	BRV15-2	W	1820	19	4	4	0
Breiðavík	BRV15-3	W	1820	19	13	13	0
Breiðavík	BRV15-4	W	1785	18	8	8	0
Breiðavík	BRV15-5	W	1680	17	8	8	0
Breiðavík	BRV15-6	W	1650	17	14	14	0
Breiðavík	BRV15-7	W	1637	17	26	26	0
Breiðavík	BRV15-8	W	970	10	19	18	0
Breiðavík	BRV15-9	W	970	10	1	1	0
Breiðavík	BRV17	W	1400	14	6	6	7
Breiðavík	BRV17	W	1410	15	27	27	28
Breiðavík	BRV17	W	1529	16	9	9	10
Breiðavík	BRV17	W	1649	17	6	6	6
Breiðavík	BRV17	W	1890	19	5	5	4
Breiðavík	BRV19	W	1450	15	21	21	17
Breiðavík	BRV19	W	1550	16	6	6	5
Breiðavík	BRV19	W	1649	17	23	22	19
Breiðavík	BRV19	W	1770	18	8	8	8
Breiðavík	BRV19	W	1785	18	2	2	2
Breiðavík	BRV19	W	1838	18	2	2	2
Breiðavík	BRV19	W	1860	19	6	6	3
Breiðavík	BRV19	W	1880	19	12	12	9
Breiðavík	BRV19	W	1900	19	11	11	2
Breiðavík	GUS13	W	1850	19	1	1	1
Gjögur	GJ	NW	1425	15	3	3	3
Grænagerði	GNR	NW	1050	11	14	14	13
Gufuskálar	GFS	W	1470	15	53	54	54
Kotið	KOT	NW	950	10	6	6	6
Kollsvík	KOV-2012	W	1710	18	10	10	0
Kollsvík	KOV-2012	W	1744	18	16	16	0
Kollsvík	KOV-2012	W	1770	18	6	6	0
Kollsvík	KOV-2012	W	1795	18	13	13	0
Kollsvík	KOV-2012	W	1820	19	3	3	0
Næfurstaðir	NFS	NW	950	10	8	12	11
Strákey	STE17	NW	1550	16	12	12	11
Útskálar	UTS	W	1850	19	5	6	6
Vatnskot	VTN	NW	925	10	30	30	29
Vatnskot	VTN	NW	987	10	4	4	4
Vatnskot	VTN	NW	1050	11	5	5	4
Vatnskot	VTN	NW	1770	18	1	1	1
